# Simple low dose radiography allows precise lung volume assessment in mice

**DOI:** 10.1038/s41598-021-83319-5

**Published:** 2021-02-18

**Authors:** Amara Khan, Andrea Markus, Thomas Rittmann, Jonas Albers, Frauke Alves, Swen Hülsmann, Christian Dullin

**Affiliations:** 1 Translational Molecular Imaging, Max-Planck-Institute for Experimental Medicine, Hermann-Rein-Straße 3, 37075 Göttingen, Germany; 2 4th Physical Institute - Solids and Nanostructures, University of Göttingen, Friedrich-Hund-Platz 1, 37077 Göttingen, Germany; 3 Institute for Diagnostic and Interventional Radiology, University Medical Center Göttingen, Robert-Koch-Straße 40, 37075 Göttingen, Germany; 4 Clinic for Hematology and Medical Oncology, University Medical Center Göttingen, Robert-Koch-Str. 40, 37075 Göttingen, Germany; 5 Clinic for Anesthesiology, University Medical Center Göttingen, Humboldtallee 23, 37073 Göttingen, Germany

**Keywords:** Biological techniques, Medical research

## Abstract

X-ray based lung function (XLF) as a planar method uses dramatically less X-ray dose than computed tomography (CT) but so far lacked the ability to relate its parameters to pulmonary air volume. The purpose of this study was to calibrate the functional constituents of XLF that are biomedically decipherable and directly comparable to that of micro-CT and whole-body plethysmography (WBP). Here, we developed a unique set-up for simultaneous assessment of lung function and volume using XLF, micro-CT and WBP on healthy mice. Our results reveal a strong correlation of lung volumes obtained from radiographic XLF and micro-CT and demonstrate that XLF is superior to WBP in sensitivity and precision to assess lung volumes. Importantly, XLF measurement uses only a fraction of the radiation dose and acquisition time required for CT. Therefore, the redefined XLF approach is a promising tool for preclinical longitudinal studies with a substantial potential of clinical translation.

## Introduction

Respiratory diseases account for ~ 10% of mortality worldwide, which is set to rise due to persistent smoking, pollution and occupational irritants^1^. Depending on the underlying disease, the characteristics of lung dysfunction may involve inadequate oxygen exchange, breath shortening, chronic cough, chest pain and dyspnoea^2,3^. To improve our knowledge about the basic mechanisms regarding lung physiology, pathophysiology and treatment strategies, the use of mouse models of lung disease in translational research is inevitable. The pre-clinical assessment of pulmonary function is a valuable tool not only for investigating the pathology of respiratory and allergic diseases but also enables preclinical evaluation of the response to novel therapeutic strategies.

Changes in lung function can be monitored non-invasively in restrained or unrestrained mice by double chamber plethysmography or barometric plethysmography^2,4^. Other methods require invasive intubation procedures some of which tend to be terminal^5^. Of all available techniques, unrestrained whole-body plethysmography (WBP) is extensively used in longitudinal studies due to its ease of use and high data output^3,6–8^. However, there is also considerable concern over the validity of plethysmography techniques^9–12^. The main limitation of this technique is that the functional outcomes rely on the plethysmograph pressure changes, and the relationship between chamber pressure and the lung mechanics might be non-linear. Furthermore, the accurate tidal volume estimates are significantly affected by subtle changes in temperature, pulmonary mechanism and fluctuations in barometric pressure^3,10,13^. Plethysmography may also be inconsistent between mouse strains requiring precise selection of the control groups^11^.

Recently, the use of lab and synchrotron radiation based micro-computed tomography (micro-CT) has been increasingly used in preclinical studies to assess quantitative parameters including lung volumes, air spaces or lesions and mean lung density^14–19^. These measurements have been performed in an attempt to reflect the structural and functional competence of the lung during disease progression and in response to therapy in numerous animal models of lung diseases, such as cancer, fibrosis, emphysema and transplantation^14^. Not only in clinical routine, but also in preclinical research, dose restrictions are major obstacles. Small-animal models, especially mouse models, are often utilized in preclinical research. Imaging in live mice for micro-CT based longitudinal lung studies requires a dramatically higher spatial and temporal resolution due to the small size of the mouse and its fast metabolism. Consequently, this limits the use of CT based high-resolution imaging for longitudinal lung studies and also hampers the clinical application of this approach^20–26^.

Therefore, the development of novel and sophisticated lung function measurement techniques with improved reliability and minimal radiation is fundamentally required. To this end, we recently established a non-invasive lung function method based on planar cinematic X-ray imaging of the chest, namely X-ray-based lung function (XLF) to measure the lung function in preclinical mouse models of allergic airway inflammation (AAI) over time^27,28^. Using minimal radiation dose and short exposure time, XLF showed significantly higher sensitivity than WBP for reliable assessment of lung function during AAI and response to dexamethasone treatment^27^. However, previously the functional parameters of XLF were not exactly relatable to commonly used lung function techniques including WBP and micro-CT and none of the XLF parameters represented the lung volume.

In this study, we compared XLF determined end-inspiratory volume (EIV) with the lung volumes from micro-CT and conventional WBP to establish XLF as a biomedically relevant technique. To achieve this, we first designed a unique experimental set-up for performing correlative lung function measurements using XLF, micro-CT and WBP. Using micro-CT as a gold standard approach, XLF was found sensitive and precise to determine lung volumes, as it exhibited a higher degree of correlation with micro-CT than WBP. By successfully correlating two-dimensional (2D) XLF measurements directly with three-dimensional (3D) micro-CT data in healthy mice, we present the refined XLF algorithm as a sensitive and efficient tool for determining lung volume at minimal radiation dose and exposure time.

## Results

### Experimental set-up facilitates correlative lung function measurements

**Figure 1:**
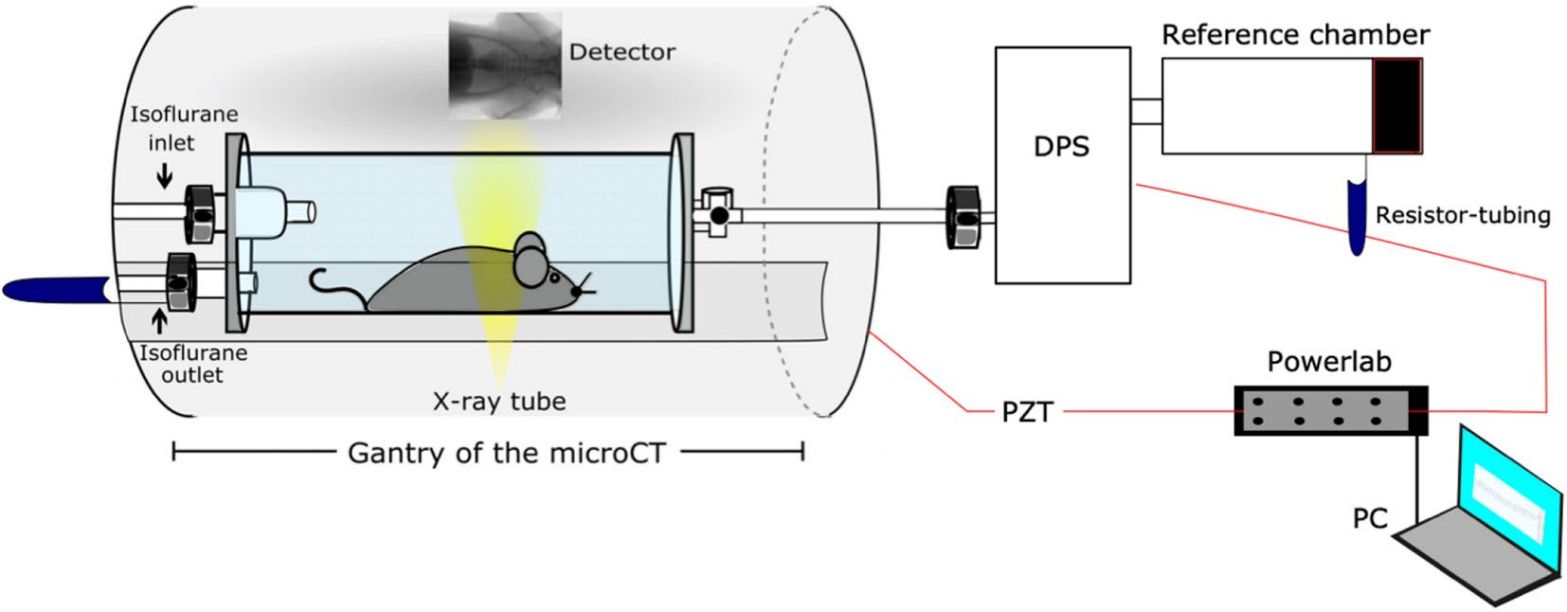
Schematic illustration of the set-up for correlative XLF, WBP and micro-CT measurements. One end of the WBP chamber has an isoflurane inlet/outlet, the other end is connected to the differential pressure sensor (DPS) which in turn is connected to a reference chamber, a Powerlab data acquisition device and a portable computer (PC). The chamber is placed inside the gantry of the micro-CT imaging system on the sample stage. The mouse is positioned inside the chamber such that the chest cavity is within the field of view (FOV). A piezoelectric (PZT) acoustic sensor that transduced the sound of the CT door interlock is used to synchronize data acquisition from WBP with XLF or micro-CT.

To correlate the XLF parameters directly with WBP and micro-CT, we designed a unique experimental set-up that enabled simultaneous measurements by combining either XLF or micro-CT with WBP. As shown in Fig. 1, the custom-made plethysmography chamber was placed into the gantry of the in-vivo micro-CT system. The chamber had multiple connectors, which served as isoflurane inlet/outlet and for connecting a differential pressure sensor (DPS; see “Materials and methods” section). Overall, the designed set-up served as an ideal platform to perform correlative lung function.

### Correlating the timing of micro-CT and WBP signal

**Figure 2:**
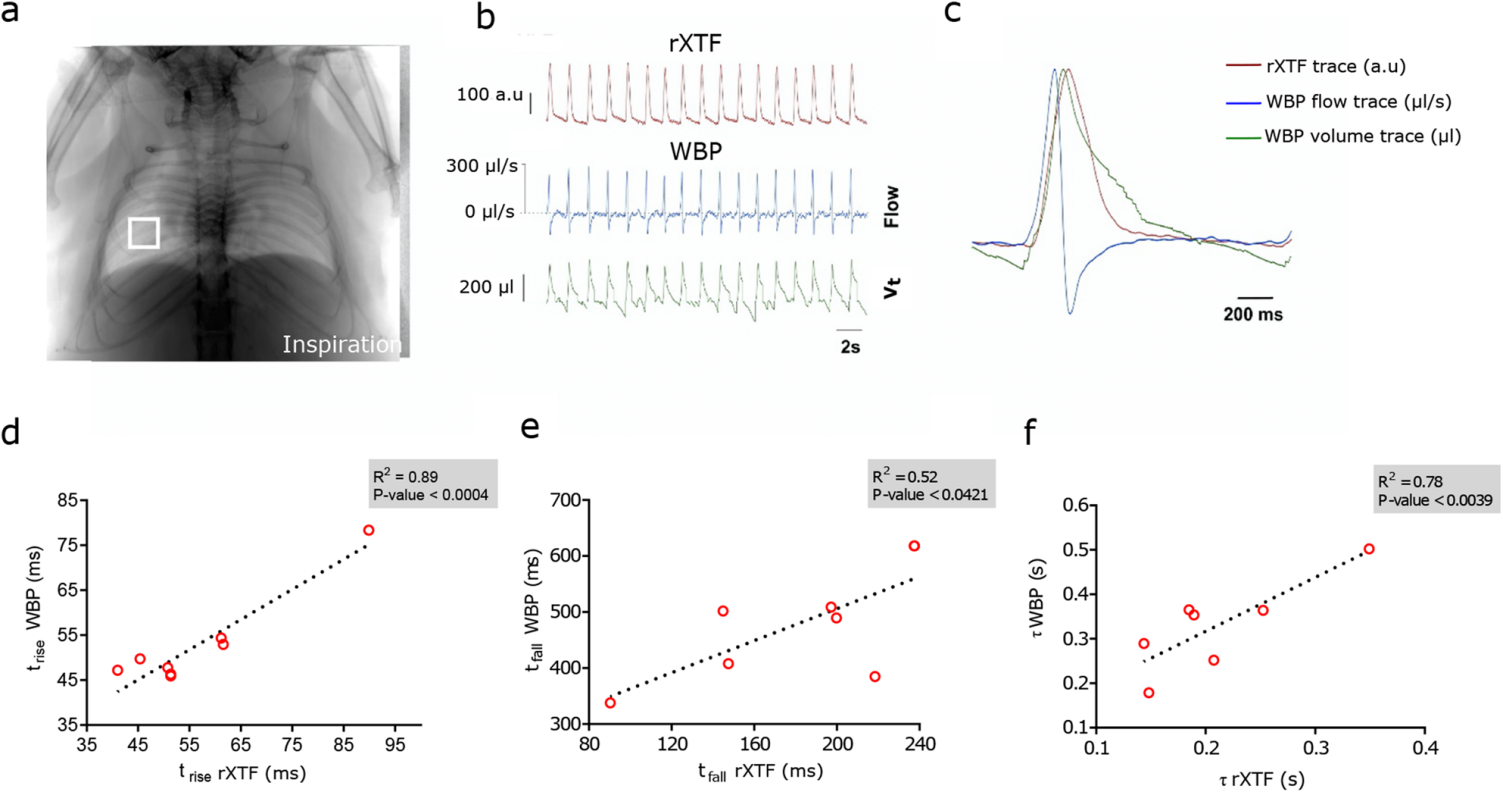
Comparison of traces from relative X-ray transmission function (rXTF) at lung region and WBP. (**a**) XLF-image in inspiration. The white box indicates the region of interest (ROI) that was used to measure changes in intensity of rXTF. (**b**) Data from rXTF and WBP. Red trace: rXTF at the ROI (arbitrary units). Blue trace: band pass filtered (0.5–20 Hz) flow trace (µl/s) from the WBP. Green trace: WBP Volume trace (V_t_, µl) derived from the integral of the flow (reset each cycle). (**c**) Averaged data from (**b**). (**d**–**f**) Correlation of the time course and signal derived from rXTF and WBP, (**d**) time rise (t_rise_) of the signal (20–80%), (**e**) decay of the signals (t_fall_) (20–80%) and (**f**) tau (τ) using a mono-exponential fit (peak to baseline, LabChart 8.0, ADInstruments). p values and respective coefficients of determination (R^2^) from linear regression analysis are shown on the graph.

To perform direct correlation between micro-CT and WBP data we tested and synchronized the timing of signal from each entity. Since the CT-scanner had no signal output to provide information about the timepoint the first image frame was acquired, we had to conceive our own trigger from the CT by installing a piezoelectric sensor to provide an acoustic signal for matching the starting point of CT data acquisition and WBP recording. The peak signals from each method were then synchronized to match the timing and traces of signals recorded. When comparing the peak of the average X-ray attenuation at the region of interest (ROI) over time (Fig. 2a) and the peak of the WBP volume signal we noticed a significant delay of 27.5 ± 20.0 ms (p < 0.01, paired t-test) (Fig. 2b,c). However, taking into consideration the exposure time of the X-ray detector of 33 ms, we assumed that the minimum X-ray attenuation at the lung region and the peak of the WBP lung volume trace coincide as expected (Supp. Fig. S1).

The rise time (t_rise_) of the rXTF signal (55.4 ± 16.1 ms) and the WBP volume signal (52.8 ± 8.7 ms) did not differ (p = 0.0004, paired t-test) (Fig. 2d). This indicates that WBP can predict the inspiratory phase of the respiratory cycle very precisely. However, while comparing t_fall_ and tau (τ) of the expiratory phase, the two methods revealed significantly different results: t_fall_ 187.1 ± 53.8 ms for rXTF versus 497.4 ± 100.8 ms for WBP (p < 0.0421; paired t-test, Fig. 2e) and τ 215.4 ± 65.4 ms for rXTF and 341.3 ± 92.6 ms for WBP (p < 0.0039, Fig. 2f). One factor that may account for the difference is the uncertainty regarding the position of the zero-flow point for the end of each respiratory cycle. Moreover, the slower flow for rXTF during expiration that accounts for this difference, might be a consequence of isoflurane-induced bronchoconstriction^29^.

### Micro-CT based volume measurements

**Figure 3:**
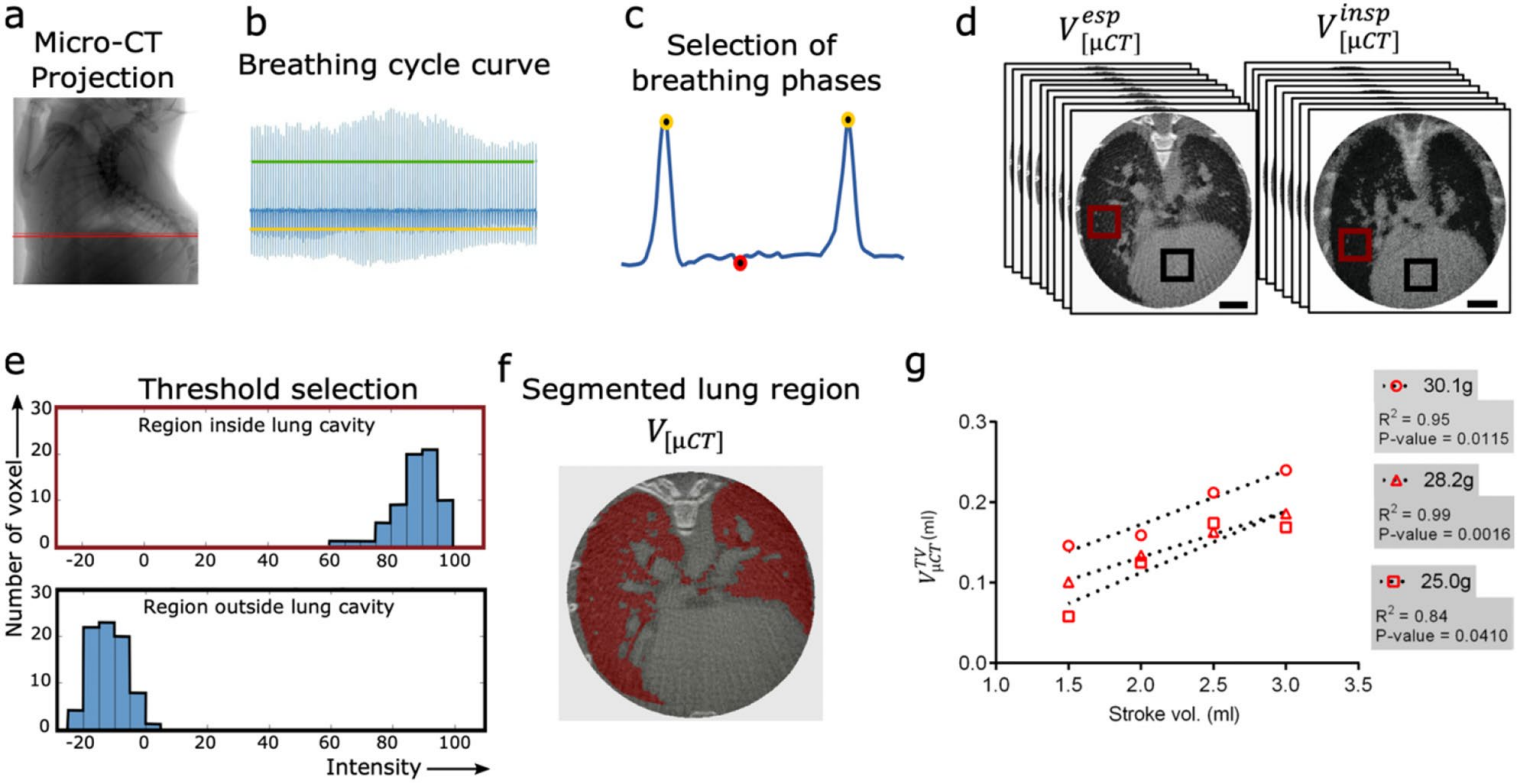
Quantification of 3D lung volumes at expiration and inspiration. (**a**) The red line marks the exemplary ROI selected near the diaphragm for representing the lung motion in the micro-CT projections. (**b**) A graph for the lung motion at the ROI is generated where the green and yellow lines show the threshold lines for maxima and minima selection, respectively. (**c**) A representative single breathing cycle is shown with an exemplary selection of one frame for expiration phase (red circle) and two frames for inspiration phase (yellow circles). (**d**) Following the selection of multiple breathing phases from the entire breathing curve, the frames from the whole micro-CT projection are sorted to reconstruct two segregated 3D lung volumes for expiration and inspiration. (**e**) A region inside and outside the lung (red and black squares in **d**) were selected to generate histograms showing the distribution of grey values for each region. (**f**) The lung volume (${V}_{\left[\mu CT\right]}$) (red) is segmented through a region growing method. (**g**) The calculated tidal volumes from micro-CT (${V}_{\left[\mu CT\right]}^{TV}$) from the segmented lung region show a strong positive correlation with the increasing stroke volumes used for mechanically ventilating euthanized mice. The body weight of the mice, p values and respective coefficients of determination (R^2^) from linear regression analysis are shown on the graph. Scale bars in (**d**) represent 1 cm.

We derived separate volumes (${V}_{\left[\mu CT\right]}$) for the lung in inspiration and expiration from micro-CT by performing retrospective gating in combination with a modified Feldkamp (FDK) reconstruction algorithm^30^. Both steps were implemented in a custom-built software called *RetrospeCT*. We used the FDK algorithm implemented in the TIGRE toolbox (https://github.com/CERN/TIGRE) which—in contrast to the typically applied version of that algorithm—allows using non-uniformly distributed angular projections. From the angular projections, an ROI line was adjusted to select the representative region of lung expansion near the diaphragm (Fig. 3a). Figure 3b shows an exemplary breathing cycle curve generated by the temporal variations in the X-ray attenuation at this ROI. A moving average filtered curve which presented gradual variations in the trendline was subtracted from the signal to factor out the background. Moreover, the minimum peak distance (expiration points) and the number of frames detected per inhaled phase were adapted to optimize the selection of projections that maintained a consistent data quality for both phases (Fig. 3c). The selection was based on thresholding: here, the highest value above the inspiration threshold (green line) and the lowest value below the expiration threshold (yellow line) were used for defining end-inspiration and end-expiration frames (Fig. 3b).

From the breathing curve, frames at the expiration and inspiration phases were selected resulting in two projection sets with strongly reduced frame numbers (approximately 340 each out of the 8892 frames recorded) and non-equidistant angular spacing (Fig. 3d). Owing to the low number of frames, standard reconstruction algorithms yielded a 3D reconstruction of poor quality. Thus, a modified FDK reconstruction algorithm based on the graphics processing unit (GPU) was used for tomographic reconstruction of the lung for both the expiration and inspiration set of projections (Fig. 3d, Suppl. Fig. S2). Still, the resulting 3D data demonstrated a high noise level and a non-uniform background intensity which hindered the segmentation of the lung and thereby a precise volume measurement. To correct the non-uniform background, a slice below the lung region was subtracted from all other slices. Subsequently, a 3D Gaussian-filter was applied to reduce the noise level (Suppl. Fig. S2). From the resulting filtered volume, the lung at both breathing phases was segmented individually. The lung was first masked to eliminate the area of the surrounding thoracic regions. To select the intensity values that enabled an optimal selection of ${V}_{\left[\mu CT\right]}$, histograms were obtained from manually defined regions inside and outside the lung cavity (Fig. 3e). A region growing algorithm was utilized in which the upper and lower threshold values from the histogram were exploited to select voxels consistent with the intensity of the aerated regions. The gaps within the resulting segmented region were filled by applying a closing operator. Lastly, ${V}_{\left[\mu CT\right]}$ was calculated from the segmented region by simply multiplying the number of segmented voxels with the volume per voxel. Other denser regions such as the mediastinum, consolidation of lung parenchyma and the airway tracts were excluded (Fig. 3f). In principle, ${V}_{\left[\mu CT\right]}$ did not only account for the air spaces within the lung but also for the micro tissue structures including small blood vessels and connective tissue which could not be segmented due to limited resolution of the reconstruction.

For testing the reliability and precision of the described workflow, ${V}_{\left[\mu CT\right]}$ measurements were performed in three dead mice of different age, weights and strains by mechanically ventilating them with increasing stroke volumes (Tables S1, S2). To achieve this, the tidal volumes from micro-CT (${V}_{\left[\mu CT\right]}^{TV}$) were determined by calculating the difference between the volume of lung cavity during inspiration and expiration (${V}_{\left[\mu CT\right]}^{insp}-{V}_{\left[\mu CT\right]}^{exp}$). The fractional increase in ${V}_{\left[\mu CT\right]}^{TV}$ correlated strongly with the applied stroke volumes (Fig. 3g). Unlike the ventilator stroke volume which represents the total ventilation volume, ${V}_{\left[\mu CT\right]}^{TV}$ measures the volume of lung aerated regions and a strong correlation between these parameters signifies that ${V}_{\left[\mu CT\right]}^{TV}$ indeed reflects the volume of air exchanged. All three mice weighing 30.1 g, 28.2 g and 25.0 g expressed a strong linear positive correlation between both variables with R^2^ values of 0.95, 0.99 and 0.84, respectively (Fig. 3g). However, the relative change between ${V}_{\left[\mu CT\right]}^{TV}$ and the stroke volume did not show an absolute match, which could be attributed to an amount of air leakage, which depends on various factors, including the speed of ventilation, increased airway resistance and reduced lung compliance in dead mouse. Nevertheless, the sensitivity of ${V}_{\left[\mu CT\right]}^{TV}$ to the changes in stroke volume warranted the use of this approach as a standard procedure for correlating the lung volumes measured by each method.

### Correlation of micro-CT determined volumes with WBP

**Figure 4:**
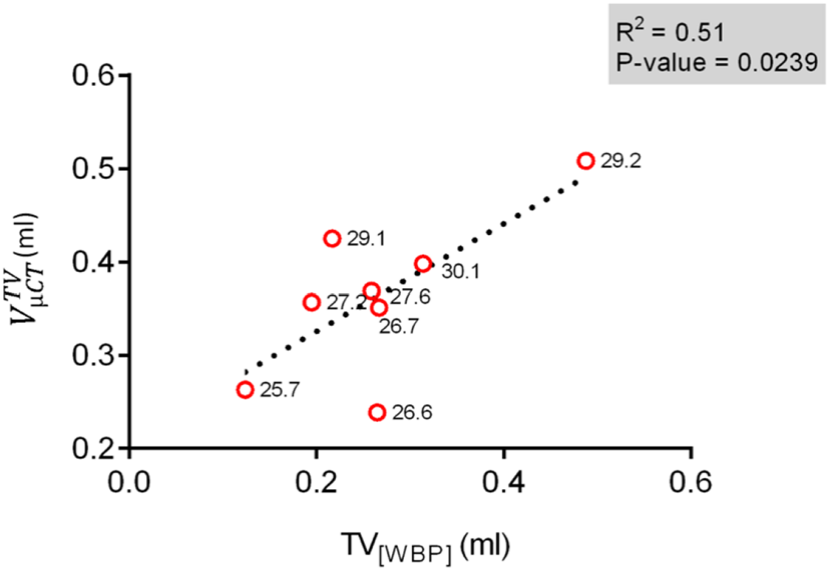
Correlation between lung volumes measured using micro-CT and WBP in living mice. Graph showing a weak positive correlation between TV_[WBP]_ with ${V}_{\left[\mu CT\right]}^{TV}$ (R^2^ = 0.5061) performed in-vivo (n = 8) using the correlative set-up. All points are labelled with the respective weights of the mice. The p values and respective coefficients of determination (R^2^) from linear regression analysis is shown on the graph.

Using the correlative approach, the in-vivo micro-CT measurements were performed in parallel with WBP. For comparing the data obtained from both methods, ${V}_{\left[\mu CT\right]}^{TV}$ was correlated with the tidal volume from WBP (TV_[WBP]_). The parameters presented only a weak correlation (correlation coefficient R^2^ = 0.5061, p value = 0.0239, Fig. 4).

Interestingly, both parameters correlated rather poorly (R^2^ = 0.5061), probably due to the fundamental differences in volumetric constituents measured by each method. While ${V}_{\left[\mu CT\right]}^{TV}$ quantifies the aerated regions of the lung, TV_[WBP]_ is derived from pressure changes produced by warming and cooling of the inspired air during every single breath^31,32^. Moreover, the volume is integrated from the calibrated flow signal (Fig. 2). Although band-pass filtering removed the bias flow, the exact zero-flow point for each respiratory cycle remains uncertain due to change of temperature and humidity inside the WBP chamber.

### Quantification of advanced XLF parameters

**Figure 5:**
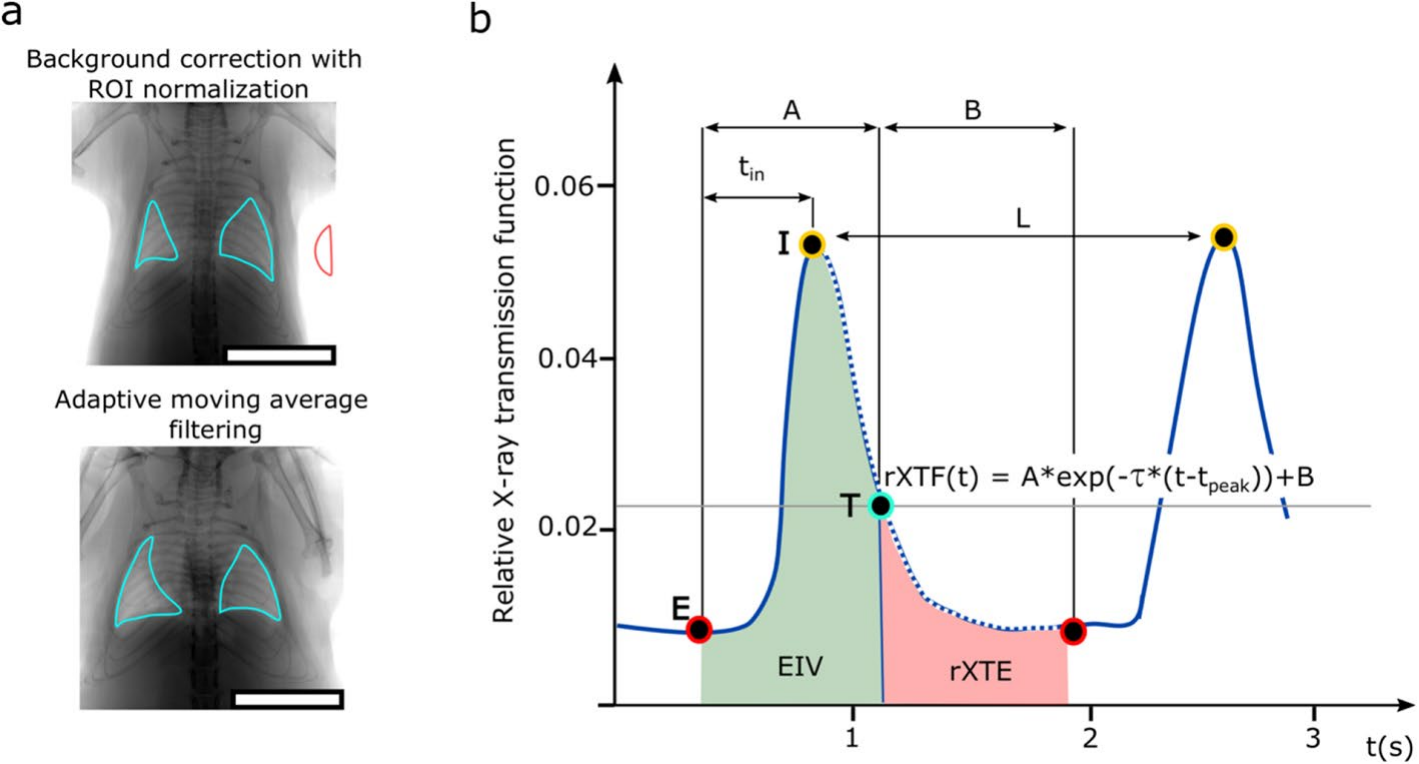
Quantification of advanced XLF parameters from the X-ray transmission over time. (**a**) A representative radiograph is shown, demonstrating background correction based on the selection of ROI at the lung lobes marked in cyan normalized by selection of the background marked in red (top panel). The modified background correction at ROI is accomplished by applying an adaptive moving average filter which requires a smaller FOV (bottom panel). (**b**) Two exemplary breathing cycles extracted from the averaged X-ray transmission at the ROI are shown for a healthy mouse over a period of 3 s. The peak intensities representing maximum inhalation **I** are marked by yellow circles while the red circles represent the beginning of a new breathing event and the maximum exhalation phase **E** of the breathing cycle. The breathing curve is split at threshold **T** (marked by a cyan circle and a grey horizontal line) to obtain a breathing (A) and a passive expiration (B) phase. XLF parameters are shown including average inhalation time (t_in_), average breathing length (L), X-ray-based end-inspiration volume (EIV_[XLF]_), relative X-ray transmission at end-expiration (rXTE) and exponential function (τ, calculated in region indicated by blue dotted line). Scale bars in (**a**) represent 1 cm.

The functional parameters for XLF were measured from the average X-ray transmission at the chest area over time. Previously, we reported the calibration of X-ray transmission over time via the selection of a ROI at left and right lung lobes at the point of maximum contraction, which was normalized by the averaged background intensity selected at a region outside the mouse^26^. However, to accomplish adequate background selection, a large FOV is required, which in turn compromises the image resolution at the lung region (Fig. 5a, top panel). Therefore, we now used a modified approach for background correction (Fig. 5a, lower panel). Briefly, an adaptive moving average filter was applied on the X-ray transmission functions **XTF(t)**, which in the first pass computed the moving average **ma(t)**, penalised each time point with its absolute distance **d(t)** to **ma(t)** and in a second pass calculated the adaptive moving average **ama(t)** resulting in a baseline of the function that is less affected by the breathing events.

$$ma\left(t\right)=\frac{1}{2n}{\sum }_{t-n}^{t+n}XTF\left(i\right)$$

$$d\left(t\right)=1/\left[\left|ma\left(t\right)-XTF\left(t\right)\right|+0.1\right]$$

$$ama\left(t\right)={\sum }_{t-n}^{t+n}d\left(i\right)\times XTF\left(i\right)/{\sum }_{t-n}^{t+n}d\left(i\right)$$

The exemplary relative X-ray transmission [rXTF(t)] shown for two breathing events in Fig. 5b, was then calculated as follows:

$$rXTF\left(t\right)=XTF\left(t\right)/ama\left(t\right)$$

The possibility to select a smaller FOV during XLF acquisition by implementing the modified background correction method led to an improved image resolution at the lung region (Fig. 5a, lower panel). This relatively enhanced macroscopic visualization of the lung structures enabled precise selection of the aerated regions at each lung lobe.

Following background correction, the calibrated X-ray transmission over the chest region was used for quantification of in-vivo XLF. The functional parameters measured by XLF were determined from the breathing curve (Fig. 5b). The local maxima **I**, which denotes the time points of maximum inspiration (yellow circles, Fig. 5b) were detected. Then the time points of maximum expiration **E** (red circles, Fig. 5b) were identified as the closest local minima before the maxima **I**. To further suppress the effect of noise a moving average filter was applied to define the minima **E**. To split the breathing curve into an active breathing (mainly inspiration) and a passive expiration phase, an intersection point was used at a level function (grey horizontal line, Fig. 5b) resulting in the threshold point rXTF **T** (cyan circle, Fig. 5b). Moreover, this point also separates the background signal and noise from the breathing peaks.

Since quantitative functional parameters in the rXTF are based on the analysis of every single breath, they were measured as an average over the entire recorded data. The inspiration time (**t**_**in**_) was calculated as the average time between the points **E** and **I** for all breathing events recorded. Further, the length of the breathing events (**L**) was calculated as the average distance between the peaks **I** of the rXTF. The first part of the area under the curve (**AUC**) in the interval **E** to **T** was recorded as end-inspiration lung volume (**EIV**_**[XLF]**_) while the second part in the interval **T** to **E** denoted the relative X-ray transmission at end-expiration (rXTE). Lastly, to characterize the decay rate **τ** of the expiration phase (represented by the blue dotted line), which indirectly measures changes in the elastic recoil of the lung, an exponential decay function

$$rXTF\left(t\right)=A\times exp\left(-\tau \times \left(t-{t}_{peak}\right)\right)+B$$

was fitted at the intervals **I** to **E**.

### XLF measurements for precise lung volumes

**Figure 6:**
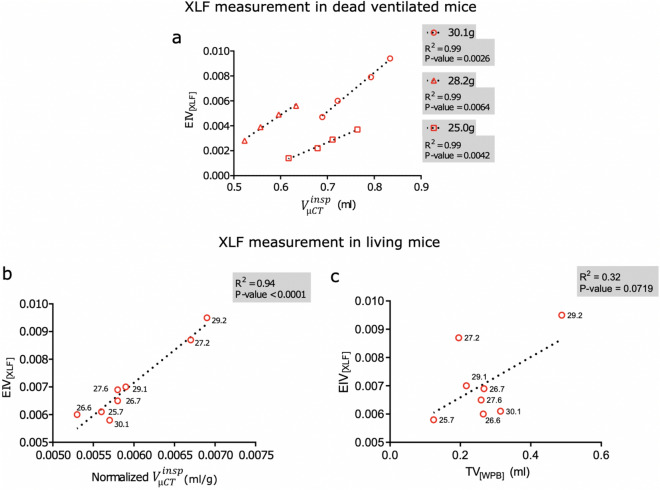
Correlation of XLF measurements with micro-CT and WBP. (**a**) Graph showing a strong positive correlation between EIV_[XLF]_ with ${V}_{\left[\mu CT\right]}^{insp}$ performed on dead mechanically ventilated mice (n = 3). (**b**) Graphs showing a strong positive correlation between EIV_[XLF]_ and ${V}_{\left[\mu CT\right]}^{insp}$ (R^2^ = 0.94) and (**c**) a weak correlation of TV_[WBP]_ with EIV_[XLF]_ (R^2^ = 0.32). Data for both b and c is shown for living mice (n = 8). The respective p values and coefficients of determination (R^2^) from linear regression analysis are shown on the graph. (**b**,**c**) All points are labelled with the respective weights of the mice.

To assess the comparability of XLF based 2D measurements with the lung volumes calculated from 3D projections, we investigated the relationship between the XLF parameters that coincided with the ${V}_{\left[\mu CT\right]}$ (Tables S2, S3). We therefore compared EIV_[XLF]_ which is described by the average AUC in the X-ray transmission function of a breathing event with ${V}_{\left[\mu CT\right]}^{insp}$. Since EIV_[XLF]_ is mostly affected by the total air volume inhaled at the end of the inspiration phase it should be strongly related to the ${V}_{\left[\mu CT\right]}^{insp}$. To analyse the correlation between the two parameters, we again assessed the lung volumes in dead mice that were ventilated with increasing stroke volumes. Since ${V}_{\left[\mu CT\right]}^{insp}$ values have a lower standard error than ${V}_{\left[\mu CT\right]}^{TV}$, they showed an improved correlation with increasing stroke volumes (Supp. Fig. S3) as compared to ${V}_{\left[\mu CT\right]}^{TV}$ (Fig. 3g). EIV_[XLF]_ also exhibited a strong positive correlation with ${V}_{\left[\mu CT\right]}^{insp}$ for all three mice (30.1 g, R^2^ = 0,99; 28.2 g, R^2^ = 0.99; and 25.0 g, R^2^ = 0,99) (Fig. 6a). Importantly, EIV_[XLF]_ also corresponded to the body weight of the dead mice, unlike ${V}_{\left[\mu CT\right]}^{insp}$ which was found to be less sensitive to the body weight of the mice (Supp. Fig. S3). This is because EIV_[XLF]_ is directly influenced by temporal variations in the X-ray transmission function during air exchange. EIV_[XLF]_ hence quantifies the air volume more precisely than ${V}_{\left[\mu CT\right]}^{insp}$ which determines the volume of the entire aerated lung regions including miniscule soft tissue structures.

Following the calibration of EIV_[XLF]_ in dead mice, we applied this approach in living mice. To establish a direct correlation coefficient between the 2D (XLF) and 3D (micro-CT) datasets in living male mice, which were age and strain matched, the ${V}_{\left[\mu CT\right]}^{insp}$ was normalized with the mouse body mass to the power of 1.5 (Table S3). In living mice, a strong linear positive correlation between EIV_[XLF]_ and normalized ${V}_{\left[\mu CT\right]}^{insp}$ was observed with a correlation coefficient of R^2^ = 0.94 (Fig. 6b). These findings revealed that lung volumes determined from XLF were precise and comparable to those measured from 3D data acquired by micro-CT. It is noteworthy that the XLF data were recorded in 34 s to determine both lung volume and function with an average X-ray dose of only 0.17 Gy. In comparison, CT data were acquired in a considerably longer acquisition time of 4.5 min and with a relatively high X-ray dose of approximately 2.7 Gy. This signifies that XLF can perform both lung volume and function measurements in substantially faster acquisition time and by using only a fraction of the X-ray dose that is necessary for CT.

Because tidal volumes cannot be derived from XLF measurements, a direct comparison of XLF and WBP measurements is not possible. Still, a correlation between EIV_[XLF]_ and TV_[WBP]_ was tested which resulted in a weak correlation as expected (R^2^ = 0.32, Fig. 6c; Table S3). Using ${V}_{\mu CT}$ as a standard approach, it can be concluded that EIV_[XLF]_ is sensitive and accurate for determining lung volumes at inspiration_._ Overall, these findings also highlight the difficulties in deriving lung volumes from WBP measurements as compared to XLF.

## Discussion

This study presents a refined XLF approach, which enables the determination of lung volumes. This was achieved by using a novel experimental set-up which facilitated correlative assessment of lung volumes in mice using XLF, micro-CT and conventional WBP. To enable the biomedical interpretation of the XLF-acquired parameters as well as their comparison with WBP and CT, XLF parameters described in Dullin et al.^26^ were redefined^27^. Our results demonstrate that lung volume derived from a simple 2D measurement (XLF) strongly correlates with the volume of the lung determined from micro-CT data.

Semi-automated segmentation of CT images is commonly used to define total lung volume in healthy and diseased mice^15,19,33–37^, hence we used this method in this study as a benchmark for correlation of lung volumes. However, poor resolution owing to low signal-to-noise-ratio and motion induced image blurring could limit the lung volume quantification using retrospectively gated CT^38^. Likewise, in the present study due to the comparably large voxel size of 40 µm and motion artefacts, it was not possible to calculate the volume of air only, instead, we measured the entire volume of the aerated regions of the lung. Moreover, changes in the morphology and compliance of the lung can occur at the early post-mortem stage, affecting the total ventilation volume reaching the lungs^16^. The extent of these post-mortem alterations may even be different in each individual mouse. This explains the lack of perfect correlation between ${V}_{\left[\mu CT\right]}$ and stroke volumes as well as the body weight of the dead mice*.* Other factors such as the relative fast stroke rate of the ventilator (40 strokes/min), leakage at the tracheal cannula and length and elasticity of the tubes can also contribute to the differences in the total air volume exchanged in mechanically ventilated mice. We used a linear increase in stroke volume which resulted in a perfect correlation with micro-CT lung volume measurements, suggesting a high reliability in ${V}_{\left[\mu CT\right]}$ measurements. Therefore, ${V}_{\left[\mu CT\right]}$ was used as a reference for our correlative approach.

For a direct correlation between the lung volumes obtained from 3D micro-CT and planar XLF, ${V}_{\left[\mu CT\right]}^{insp}$ was normalized by an empirically estimated factor of body mass to the power of 1.5. In the present in-vivo study we used mice of the same age, sex and strain; hence it cannot be excluded that additional normalization parameters may be required to compensate for these factors. However, as most mouse studies are typically performed on gender and age matched cohorts, our XLF approach is generally applicable.

Most importantly, the XLF data were recorded with a significantly lower X-ray dose and about 87% shorter acquisition time when compared to CT (see “Results”). This signifies that by using only approximately 7% of the X-ray dose used in CT scans, XLF not only measures the lung function more precisely than commonly applied WBP but also determines the lung volume, a parameter that until now could only be reliably assessed by CT. This can serve as a breakthrough for facilitating the use of XLF in longitudinal studies on mouse models of lung disease with repeated lung function measurements without the risk of potential radiation damage to the lung.

XLF-defined lung volume only accounts for air volume at end-inspiration and cannot quantify end-expiration and tidal volumes. This contributed to the poor correlation of EIV_[XLF]_ with TV_[WBP]_$.$ On the other hand, slight breath to breath changes in the humidity and temperature inside the chamber can largely influence the tidal volumes obtained from WBP in small animals^10,39^. Unfortunately, the humidity and temperature change inside the chamber could not be recorded during correlative lung function measurements as the data logger device can interfere with the X-ray transmission and more importantly changes the posture of the mouse which can affect the breathing pattern. Consequently, we could not determine a correction factor offline (see “Materials and methods”) for the WBP tidal volume which resulted in the rather weak correlation with ${V}_{\left[\mu CT\right]}^{TV}$. Moreover, the standard errors involved in the segmentation of the lung volume at each inspiratory and expiratory phase both contribute to the resulting ${V}_{\left[\mu CT\right]}^{TV}$ values, therefore reducing its precision and accuracy.

In addition, WBP is complicated to perform in both pre-clinical and clinical use, and can result in significant inconsistencies due to minor technical differences in equipment, handling and surroundings^40,41^. Henceforth, sophisticated correlative techniques such as presented in this study are required to further test the validity of plethysmography measurements for determining tidal volumes. On the other hand, WBP for preclinical application can be performed in freely moving animals, while XLF requires isoflurane anaesthesia.

In conclusion, we present XLF as a tool for an efficient and reliable assessment of lung function and also lung volumes by successfully correlating radiographic XLF measurements with micro-CT data. Due to the high sensitivity of XLF and the ability to use standard micro-CT equipment or even a simple X-ray tube to perform these studies, XLF can easily be applied in preclinical studies. Most importantly, XLF is performed with minimal X-ray dose and acquisition time and is therefore suitable for longitudinal studies. This also provides a vital basis for clinical translation. X-ray based lung function analysis—especially for studying lung motion—has already been used, but due to X-ray dose limitations has not been routinely applied in the clinic. Fouras et al.^42^ developed a similar approach for low dose X-ray velocimetry for patient setup. However, to date, no clinical data from this system has been published. Our data, showing the use of low X-ray dose for lung function and volume measurements, support the notion that this technique should be further developed for patients. Since the functional and volumetric parameters measured by XLF are designed to reflect airway remodelling contributing to the airflow obstruction, XLF could be used for assessing multiple pulmonary diseases and even enable an early-stage diagnosis in case of pulmonary fibrosis and edema^43,44^. Ventilation settings which are currently adjusted manually by the operator and by interpreting ventilator-derived parameters could also be regularly monitored by XLF and adjusted to personalized, case-by-case settings^45^. Lastly, XLF may be potentially performed in non-compliant or even unconscious patients, in which e.g., spirometry typically used for lung function measurement, cannot be applied.

## Material and methods

### Animal experiment

Eight healthy male C57Bl/6 mice (13 weeks old) were used for in-vivo lung function measurements (Table S1). Additional three male mice were used for mechanical ventilation experiments (Table S1). The mice were kept in a temperature and humidity-controlled room with a 12 h light–dark cycle and were fed food and water ad libitum. They were euthanized by cervical dislocation. All animal procedures were performed in compliance with the guidelines of the ARRIVE, European Directive (2010/63/EU) and the German ethical laws and were approved by the administration of Lower Saxony, Germany (approval number G15.1747).

### Experimental set-up

The experimental set-up was designed to perform either XLF or micro-CT with WBP in parallel (Fig. 1). The custom-made plethysmography chamber (see below) of approximately 14 cm in length and 5 cm in diameter with a capacity of 200 ml was built to fit into the gantry of the in-vivo micro-CT system (QuantumFX, PerkinElmer) allowing acquisition of a 20 × 20 mm^2^ FOV for XLF and micro-CT imaging. The mouse was positioned in the WBP chamber, which was then placed on the sample stage inside the micro-CT. The position of mouse and stage was adjusted to have the chest-cavity within the FOV. A piezoelectric sensor (PZT, FT31T-1.3A1-472; KEPO, Ningbo, China) was installed at the door of the micro-CT device to synchronize the data acquisitions for WBP with the acoustic signal from the door interlock system at the beginning and at the end of XLF or micro-CT recording (Supp. Fig. S1).

### In-vivo XLF measurement

XLF was performed on unrestrained mice that were isoflurane-anesthetized (~ 2% isoflurane in 1 L oxygen per min) by acquiring planar cinematic X-ray images of the mice chest cavity using a low-dose in-vivo micro-CT as previously described by Dullin et al*.*^21^ and Markus et al*.*^22^. Briefly, mice were placed inside a plastic chamber (Fig. 1) and the breathing frequency of the mice was adjusted manually by changing the isoflurane concentration between 1.5 and 3% to achieve a breathing frequency of approximately 0.7 Hz. This resulted in a total of 21 breathing cycles recorded in ~ 34 s scans. XLF measurements were recorded using an X-ray tube voltage of 90 kVp and a tube current of 100 µA. The radiographs of the chest movements during the breathing process were sequenced to produce a movie, which later on was parametrized at the lung regions.

### Micro-CT imaging

In-vivo 3D micro-CT scans were made immediately after combined XLF and WBP measurements on the isoflurane-anesthetized mice. Mouse lungs were scanned by applying a cardiac gating technique using the following parameters: X-ray tube voltage 90 kVp and tube current 200 µA. The radiograph projections were taken for the 360° gantry rotation for a total scan time of 270s and the entire radiograph was gated retrospectively.

### XLF and micro-CT measurements in mechanically ventilated dead mice

Three male mice were euthanized using cervical dislocation technique. Tracheotomy was performed to ventilate the mice with increasing stroke volumes ranging from 0.5 to 3.0 ml and a fixed stroke rate of ~ 40 stokes/min using a MiniVent ventilator (Type 845, Harvard Apparatus, Holliston, USA). However, due to faster breathing in the ventilated mice, the breathing frequency was approximately one single breathing event per 860 ms. The XLF measurements and micro-CT imaging were performed as described for the in-vivo experiments.

### Unrestrained whole-body plethysmography

We used a chamber in the so-called flow-through configuration, which utilizes the principle of a pneumotach^46,47^. In detail, the pressure difference between the recording chamber (220 ml) and a reference chamber (50 ml) was captured by a differential pressure sensor (DPS, Board Mount Pressure Sensor, 0–1" H_2_O, 20 mV, 16 VDC supply; Mfr. No: INCH-D-MV, Amphenol Cooperation, Wallingford, CT 06492). Digitization was performed with an analog–digital interface (PowerLab) and LabChart-software (ADInstruments LTD, Oxford, United Kingdom). The connectors on the lid of the chamber were used for isoflurane inlet/outlet and for connecting the DPS. A positive bias flow of 0.4–0.7 L/min was introduced using the anesthesia unit (VisualSonics VS 4112; 50% Air + 50% O_2_) to supply the volatile anesthetic isoflurane. The time constant of the pressure decay from the chamber system was adjusted to approximately 40 ms to match commercial chamber systems that we used in earlier papers^47^. The decay time constant was tested by manually obstructing the air outlet and measuring the decay of the pressure signal after releasing the obstruction (LabChart Peak Analysis Module).

The raw flow signal was bandpass filtered off-line (0.5–20 Hz), to remove movement artifacts, noise and also bias flow, and then integrated for measuring a tidal volume. We used the standard integral settings of the “Integral Channel Calculation module” of the LabChart-software (use of all data points, reset each cycle, whereby the integral is reset each time the source signal passes through zero to a positive value). Temperature and humidity inside the plethysmography chamber were measured by using a LOG32 data logger (Dostmann electronic GmBH, Wertheim-Reicholzheim, Germany).

### WBP temperature and humidity measurements

CT induced temperature and humidity changes inside the plethysmography chamber could not be measured online. We therefore approximated the values for humidity and temperature correction based on separate measurements, that were performed under similar conditions to those used during correlative lung function measurements. In short, the data logger device was placed inside the chamber together with the mouse under isoflurane anesthesia and the temperature and humidity was recorded for ~ 4 min. The relative humidity of the air/O_2_ mix was below 3%. Relative humidity (rh) inside the chamber dropped during the recording. With the mouse inside the chamber, the rh ranged between 22.5 and 9.4% (mean 16.7%). Considering that the data logger reduces the volume in the chamber, the value inside the chamber without the data logger is expected to be even smaller. The average temperature inside the chamber with the mouse was 27 °C. Since the duration of anesthesia was below 15 min, we did not anticipate any significant change in the body temperature (36.5 °C). With an assumed atmospheric pressure of 760 mmHg (1.01308 bar), the volume adjustment factor was 11.7.

### WBP data sampling and analysis

Flow signal from the pressure sensor was sampled at 1 kHz, while the PTC signal was sampled at 40 Hz. The intensity of the lung was detected by using the “Plot Z-axis Profile” function of ImageJ software^48^. Therefore, an ROI was set to the level of the diaphragm in expiration. Data was exported as ACSII and imported to LabChart 8.0 (ADInstruments). For removal of low-frequency intensity changes resulting from the X-ray tube, “the smoothed” intensity trace (median filter; 101 samples = 100 ms) was subtracted from the raw trace. Processed image data and the WBP data was exported to Axon PCLAMP file format and imported into IGOR Pro 6.37 (Wavemetrics, Lake Oswego, US) for resampling (1 kHz) and reimported into LabChart for final offline analysis. The peak detection module of LabChart was used to identify respiratory parameters including tidal volume (integrated flow), T_start_ and T_end_ (10–90% of peak height) as well as the decay of the signal (τ; 100 to 0% of peak).

### Data quantification and statistical analysis

The data of lung volumes were quantified using *RetrospeCT* (https://gitlab.gwdg.de/thomas.rittmann/retrospective_gating) which includes an iterative GPU-based reconstruction toolbox (tigre, https://github.com/CERN/TIGRE). Quantification of XLF measurements were achieved with a custom-built software *xLFinal* https://github.com/xPITcoding/xLFinal.git. WBP was performed with an analog–digital interface (PowerLab) and LabChart-software (ADInstruments). For statistical analysis, the paired t-test for same mean implemented in MS Excel 2013 was used with a p value of 0.05 (*) as margin for statistical significance. To measure correlation, the Pearson correlation coefficient implemented in GraphPad Prism Software version 8 was used (GraphPad Software, La Jolla California USA).

### Ethical approval

All animal procedures were performed in compliance with the guidelines of the ARRIVE, European Directive (2010/63/EU) and the German ethical laws and were approved by the administration of Lower Saxony, Germany (approval number G15.1747).

## Supplementary Information

Supplementary Information

## Data Availability

All data generated or analyzed during this study are included in this published article and its supplementary information files.
