# Supplementary material for: Simple low dose radiography allows precise lung volume assessment in mice
Source: Sci Rep. 2021 Feb 18;11:4163. doi: 10.1038/s41598-021-83319-5 (PMC7893164; doi:10.1038/s41598-021-83319-5)
Supplement: Supplementary file 1 — Supplementary Information [file 41598_2021_83319_MOESM1_ESM.docx]

**Simple low dose radiography allows precise lung volume assessment in mice**

Amara Khan^1^, Andrea Markus^1^, Thomas Rittmann^2^, Jonas Albers^1,3^, Frauke Alves^1,3,4^, Swen Hülsmann^5, ‡^ and Christian Dullin^1,3,6 ‡^

^1^Translational Molecular Imaging, Max-Planck-Institute for Experimental Medicine, Hermann-Rein-Straße 3, 37075 Göttingen, Germany;

^2^4th Physical Institute - Solids and Nanostructures, University of Göttingen, Friedrich-Hund-Platz 1, 37077 Göttingen, Germany;

^3^Institute for Diagnostic and Interventional Radiology, University Medical Center Göttingen, Robert-Koch-Straße 40, 37075 Göttingen, Germany;

^4^Clinic for Hematology and Medical Oncology, University Medical Center Göttingen, Robert-Koch-Str. 40, 37075 Göttingen, Germany;

^5^Clinic for Anesthesiology, University Medical Center Göttingen, Humboldtallee 23, 37073 Göttingen, Germany.

***** Correspondence: christian.dullin@med.uni-goettingen.de

^†^ Shared senior authorship

**Supplementary material**

**S1: Synchronizing the timing of signals**

**Figure S1: Synchronizing x-ray and WBP based measurements.** a) X-ray and whole-body plethysmography (WBP) acquisitions were synchronized using an mechano-acoustical signal from the interlock system of the computed tomography (CT) device that was detected by a piezosensor (PZT) that was placed at the door of the CT (red trace). The delay between this signal and the first image of the radiographic image series was measured by the parallel recording of the CT images and a transistor-transistor logic (TTL) signal (dark blue trace) triggering a custom-made mechanical lever (image series), which was placed inside the CT. The TTL was generated by the Powerlab (8/35) that recorded the piezo signal. b) The response time of the lever to the TTL signal (red trace) was measured by a light reflex (blue trace) that was detected with a Fiberoptometer II (NPI electronic GmbH). For timing the 5% threshold of the signal was used.

The signals from x-ray and WBP measurements were synchronized as shown in Figure 1. The delay (CT-delay in Fig. S1a) from the click to the first image was found to be 3243.3 ± 28.4 ms (n=4). Thus, thetiming error (< 30 ms) is small then the duration of a single XLF-image (33 ms), allowing us to record the timing of the respiratory signal with sufficient precision during the entire duration of radiographic recording.

**S2**: **Animal data:**

The mice used for in-vivo assessment of lung function using the correlative approach were all age, strain and gender (male) matched. This was done to reduce the number of factors that may influence the lung function measurements. The mechanically ventilated dead mice were only gender matched (male) and therefore of different age and strains.

Table S1: Data for the mice including age, body weights and normalized body weights.

| Experiment | Mouse | Age  (weeks) | Gender | Strain | Body weight  (g) | Normalized body weights  (g^1.5) |
| --- | --- | --- | --- | --- | --- | --- |
| In-vivo | 1 | 13 | Male | C57BL/6 | 30.1 | 165.1 |
|  | 2 | 13 | Male | C57BL/6 | 27.6 | 145.0 |
|  | 3 | 13 | Male | C57BL/6 | 29.2 | 157.8 |
|  | 4 | 13 | Male | C57BL/6 | 29.1 | 157.0 |
|  | 5 | 13 | Male | C57BL/6 | 27.2 | 141.9 |
|  | 6 | 13 | Male | C57BL/6 | 25.7 | 130.2 |
|  | 7 | 13 | Male | C57BL/6 | 26.6 | 137.1 |
|  | 8 | 13 | Male | C57BL/6 | 26.7 | 138.0 |
| Mechanically ventilated dead mice | 1 | 49 | Male | BALB/c | 30.1 | 165.1 |
|  | 2 | 10 | Male | C57BL/6 | 25.0 | 125.0 |
|  | 3 | 10 | Male | C57BL/6 | 28.2 | 149.8 |

**S3: XLF, micro-CT and WBP measurements in dead ventilated mice**

Table S2: Lung volumes obtained from XLF and micro-CT in mechanically ventilated mice

|  |  | XLF | Micro-CT | |
| --- | --- | --- | --- | --- |
| Mouse | Stroke volume (ml) | EIV_[XLF]_ | $V_{[\mu CT]}^{insp}$(ml) | ${LV}_{[\mu CT]}^{exp}$(ml) |
| 1 | 1.5 | 0.0047 | 0.689 | 0.543 |
|  | 2.0 | 0.0060 | 0.722 | 0.563 |
|  | 2.5 | 0.0079 | 0.793 | 0.581 |
|  | 3.0 | 0.0094 | 0.834 | 0.594 |
| Mean ± SD | - | 0.0070 ± 0.0021 | 0.7595 ± 0.0660 | 0.57025 ± 0.0222 |
| 2 | 1.5 | 0.0014 | 0.617 | 0.559 |
|  | 2.0 | 0.0022 | 0.679 | 0.554 |
|  | 2.5 | 0.0029 | 0.711 | 0.537 |
|  | 3.0 | 0.0037 | 0.764 | 0.595 |
| Mean ± SD | - | 0.0025 ± 0.0010 | 0.6928 ± 0.0615 | 0.5613 ± 0.0244 |
| 3 | 1.5 | 0.0028 | 0.523 | 0.422 |
|  | 2.0 | 0.0039 | 0.557 | 0.423 |
|  | 2.5 | 0.0049 | 0.596 | 0.433 |
|  | 3.0 | 0.0056 | 0.633 | 0.447 |
| Mean ± SD | - | 0.0043 ± 0.0012 | 0.5773 ± 0.0477 | 0.4313 ± 0.0116 |

EIV_[XLF]_ = XLF measured end-inspiratory volume; $V_{[\mu CT]}^{insp}$ = micro-CT measured lung volume at inspiration; ${LV}_{[\mu CT]}^{exp}$ = micro-CT measured lung volume at expiration

Table S3: Lung volumes obtained from XLF, micro-CT and WBP measurements in living mice

|  | **XLF** | **Micro-CT** | | | | **WBP** |
| --- | --- | --- | --- | --- | --- | --- |
| Mouse | EIV_[XLF]_ | $V_{[\mu CT]}^{insp}$(ml) | Normalized  $V_{[\mu CT]}^{insp}$(ml/g) | $V_{[\mu CT]}^{exp}$(ml) | $V_{\left[ \mu CT \right]}^{TV}$(ml) | TV_[WBP]_ (ml) |
| 1 | 0.0061 | 0.921 | 0.0056 | 0.523 | 0.398 | 0.314 |
| 2 | 0.0065 | 0.837 | 0.0058 | 0.468 | 0.369 | 0.259 |
| 3 | 0.0095 | 1.088 | 0.0069 | 0.580 | 0.508 | 0.488 |
| 4 | 0.0070 | 0.932 | 0.0059 | 0.507 | 0.425 | 0.217 |
| 5 | 0.0087 | 0.944 | 0.0067 | 0.587 | 0.357 | 0.195 |
| 6 | 0.0058 | 0.737 | 0.0057 | 0.474 | 0.263 | 0.124 |
| 7 | 0.0060 | 0.725 | 0.0053 | 0.486 | 0.239 | 0.265 |
| 8 | 0.0069 | 0.800 | 0.0058 | 0.449 | 0.351 | 0.267 |
| Mean (±) SD | 0.0071 ± 0.001 | 0.8730 ± 0.122 | 0.0060  ± 0.001 | 0.5092 ± 0.051 | 0.3638 ± 0.086 | 0.2661 ± 0.106 |

Mean ± standard deviation (SD) is shown for all datasets. EIV_[XLF]_ = XLF measured end-inspiratory volume; $V_{[\mu CT]}^{insp}$ = micro-CT measured lung volume at inspiration; ${LV}_{[\mu CT]}^{exp}$ = micro-CT measured lung volume at expiration; $V_{\left[ \mu CT \right]}^{TV}$ = micro-CT measured tidal volume; TV_[WBP]_ = WBP measured tidal volume.

**S4: Comparison of three-dimensional (3D) data reconstructed by using the standard algorithm and modified Feldkamp (FDK) reconstruction**

The proprietary retrospective gating software of the device can only be used to remove breathing artifacts (Fig. S2a) but cannot reconstruct the lung in two separate states. To achieve a reconstruction with strongly reduced breathing artifacts the software uses 494 frames out of the 7904 initially acquired. In order to reconstruct the lung in the two states i.e., an end-inspiration and end-expiration, we used custom-made software in combination with 200 projections for each phase. Our software uses a Feldkamp algorithm that can deal with non-uniformly angularly distributed projection data and is implemented in TIGRE. This resulted in reconstruction of images shown in figure S2b, while the FDK reconstruction after the subtraction of a slice outside the lung region and following Gaussian low-pass filtering is displayed in figure S2c. The normalized profile plot (Fig. S2d) at the position indicated by the blue line over the lung region (Fig. S2a-c) shows an apparent shading in the original FDK data, which would not enable segmentation of the lung with a threshold-based algorithm. In contrast to that, the proposed subtraction of a slice outside the lung region and the subsequent Gaussian low pass filtering removes the shading and boosts the contrast to noise ratio from initially about 0.5 to 3. Subsequently, the lung region can be segmented using the threshold indicated by the gray line (Fig. S2d).

**Figure S2: Comparison of micro-CT data reconstructions using standard retrospective gating software, modified FDK algorithm and FDK-reconstruction post filtering.** a) Micro-CT reconstruction using the standard device-based algorithm with combined breathing states (inspiration and expiration) b) FDK-reconstructed image without additional correction factors. c) Image reconstruction using the modified FDK algorithm with post-process corrections including shading correction and enhanced contrast-to-noise ratio. d) The normalized profile-plot at the position indicated by the blue line over the lung region in a-c.

**S5: Correlation between micro-CT determined lung volumes at inspiration and stroke volumes in dead ventilated mice**

**Figure S3: Correlation between** $V_{[\mu CT]}^{\mathrm{insp}}$ **and stroke volumes applied for ventilating dead mice.** Graph showing strong correlation between $V_{[\mu CT]}^{\mathrm{insp}}$ and increasing stroke volume used for ventilating dead mice_._ The weights of the mice, respective coefficients of determination (R^2^) from linear regression analysis and p-values (GraphPad Software) are shown on the graph.
